# Chronic granulomatous disease: Clinical, microbial, and genetic findings in 39 Colombian patients

**DOI:** 10.70962/jhi.20250257

**Published:** 2026-05-21

**Authors:** Julian Rojas, Carlos A. Arango-Franco, Marcela Moncada-Velez, Diana Marcela Arboleda, Edgar Alfonso Figueredo, Manuela Molina, Juan Pablo Sánchez, Lina Marcela Zapata, Paula Catalina Diaz, Jesús Armando Álvarez, Maria S. Serna-Arbelaez, Lizet Jazmin Pérez Zapata, Gabriel Vélez, Camille Soudée, Juan F. Alzate, Felipe Cabarcas, Catalina Obando Gil, Carlos Garcés, Martha I. Alvarez-Olmos, Paola Marcela Pérez-Camacho, Diego Medina-Valencia, Jaime Alberto Patiño-Niño, Juan Pablo Rendón, María Claudia Ortega-López, Luz Elena Cano, Juan Francisco López, Flor Marcela Estupiñán, Mauricio Chaparro, Laura Niño, Andrés Felipe Escobar, Jean-Laurent Casanova, Stéphanie Boisson-Dupuis, Anne Puel, Carlos Olmos, Luis Miguel Sosa, Patrick Eliana Sarmiento-Wilches, César Muñoz, Julio Cesar Orrego, Juan Álvaro López, Manuela Olaya-Hernández, Natalia Builes, Estefanía Vásquez-Echeverri, Natalia Vélez-Tirado, José Luis Franco, Lina María Castaño-Jaramillo, Jacinta Bustamante, Andrés Augusto Arias

**Affiliations:** 1 Inborn Errors of Immunity Group, Department of Microbiology and Parasitology, School of Medicine, University of Antioquia, Medellín, Colombia; 2 Laboratory of Human Genetics of Infectious Diseases, Necker Branch, INSERM U1163, Paris, France; 3 Medicine Program, School of Health Sciences, Uniremington University Corporation, Medellín, Colombia; 4 St. Giles Laboratory of Human Genetics of Infectious Diseases, The Rockefeller University, New York, NY, USA; 5 Imagine Institute, Paris Cité University, Paris, France; 6 National Center for Genome Sequencing, School of Medicine, University of Antioquia, Medellín, Colombia; 7 Pablo Tobón Uribe Hospital, Medellín, Colombia; 8 Grupo Pediaciencias, Universidad de Antioquia, Medellín, Colombia; 9 Pedriatric Infectious Diseases, Fundacion Cardioinfantil, El Bosque University, Bogotá, Colombia; 10 Pediatric Allergology Service, Fundación Valle Del Lili, Cali, Colombia; 11 Medical and Experimental Mycology Group, Corporación para Investigaciones Biológicas, Medellin, Colombia; 12 Hospital Infantil Universitario San José, Bogotá, Colombia; 13 Pediatric Infectious Diseases, HOMI Misericordia Foundation Hospital, Bogota, Colombia; 14 Pedriatic Hematopoietic Stem Cell Transplantation, HOMI Misericordia Foundation Hospital, Bogota, Colombia; 15 Pedriatic Hematopoietic Stem Cell Transplantation, Pablo Tobón Uribe Hospital, Medellín, Colombia; 16 Howard Hughes Medical Institute, New York, NY, USA; 17 Department of Pediatrics, Necker Hospital for Sick Children, Assistance Publique-Hôpitaux de Paris (AP-HP), Paris, France; 18 Cayro IPS, Bogotá, Colombia; 19 Pediatric Department, Industrial University of Santander, Bucaramanga, Colombia; 20 Nationwide Children’s Hospital, Columbus, OH, USA; 21 Napoleon Franco Pareja Children’s Hospital, Cartagena, Colombia; 22 School of Microbiology, Universidad de Antioquia, Medellín, Colombia; 23 Fundación Valle Del Lili, Cali, Colombia; 24 Facultad de Medicina, Universidad EIA, Envigado, Colombia; 25 Unidad Médica Quirúrgica Otorrinolaringología, Bogotá, Colombia; 26 Pediatric Immunology, HOMI Fundación Hospital Pediátrico La Misericordia, Bogotá, Colombia; 27 Cellular and Molecular Immunology Group, El Bosque University, Bogota, Colombia; 28 Center for the Study of Primary Immunodeficiencies, Necker Hospital for Sick Children, AP-HP, Paris, France

## Abstract

Chronic granulomatous disease (CGD) is an inborn error of immunity of caused by pathogenic variants of genes encoding components of the phagocyte NADPH oxidase complex, resulting in defective reactive oxygen species production and impaired microbial killing. We conducted a multicenter evaluation of 39 Colombian patients with CGD from 32 unrelated kindreds, describing their clinical, microbiological, and genetic characteristics. Genetic analyses were performed for 31/39 patients and identified variants of the following genes: *CYBB* (*n* = 22), *CYBA* (*n* = 3), *NCF1* (*n* = 1), *NCF2* (*n* = 1), and *NCF4* (*n* = 4). All but three of the patients had symptoms, the exceptions being individuals with p40^*phox*^ deficiency. BCG-related complications occurred in eight patients, pulmonary tuberculosis in four, and *Salmonella* spp. bacteremia in 12 of 17 patients with *Salmonella* infections. Colombian patients with CGD had clinical and microbiological profiles similar to those reported across Latin America. The genetic findings broaden the regional variant spectrum and emphasize the need for earlier diagnosis and better access to specialist testing.

## Teaser

CGD is an inborn error of immunity caused by pathogenic variants in genes encoding the phagocyte NADPH oxidase complex. In a multicenter cohort of 39 Colombian patients, we define clinical, microbiological, and genetic features, expanding the regional variant spectrum and highlighting the need for earlier diagnosis and improved access to specialized testing.

## Graphical Abstract

**Figure:**
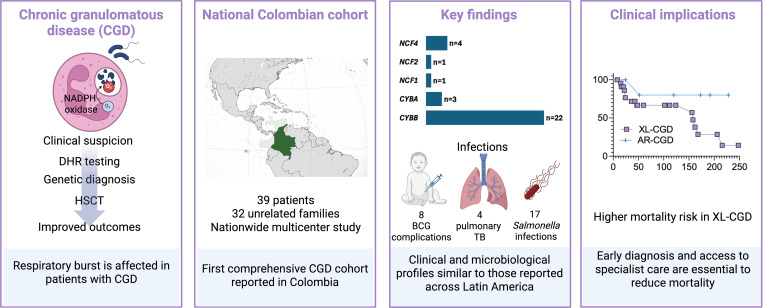

## Introduction

Chronic granulomatous disease (CGD) is an inborn error of immunity (IEI) characterized by abolished or impaired activity of the nicotinamide adenine dinucleotide phosphate (NADPH) oxidase complex, which is essential for the generation of reactive oxygen species (ROS) and microbial killing. The clinical manifestations of CGD typically appear during infancy or early childhood (1). Patients with CGD are particularly susceptible to *Staphylococcus aureus*, *Burkholderia cepacia*, *Escherichia coli*, *Nocardia* spp., *Pseudomonas* spp., *Serratia marcescens*, *Salmonella* spp., and *Klebsiella* spp. Infectious diseases due to *Mycobacterium tuberculosis*, the mycobacterium responsible for tuberculosis, or *Mycobacterium bovis*–Bacille Calmette-Guérin (BCG) vaccine have also been documented in CGD patients. The organs most frequently affected are the lungs, lymph nodes, liver, bones, gastrointestinal tract, and skin (2, 3). Fungal infections are also frequent and mostly caused by filamentous molds. Invasive aspergillosis due to *Aspergillus fumigatus* is frequently documented, followed by infections due to *Aspergillus nidulans*, *Aspergillus terreus*, and *Aspergillus tanneri* (4, 5). In addition to this susceptibility to infection, CGD patients display a predisposition to the development of granuloma and dysregulated chronic inflammation (1, 4, 5, 6, 7, 8, 9). CGD can be diagnosed biologically through assays evaluating NADPH oxidase function, including the nitroblue tetrazolium test, ferricytochrome-*c* reduction, and various chemiluminescent or fluorescent probe–based methods. The dihydrorhodamine 123 (DHR) assay, a fluorescence-based test quantifying hydrogen peroxide production and performed by flow cytometry, has become the preferred method in routine clinical laboratory diagnosis (10).

Genetically, CGD results from loss-of-function or rare hypomorphic variants of any of the genes encoding components of the phagocyte NADPH oxidase complex. This multicomponent enzymatic complex includes three cytosolic subunits—p47^*phox*^, p67^*phox*^, and p40^*phox*^—encoded by *NCF1*, *NCF2*, and *NCF4*, respectively; two membrane-bound components—gp91^*phox*^ and p22^*phox*^—encoded by *CYBB* and *CYBA*, respectively; and the chaperone protein EROS (encoded by *CYBC1/C17ORF62*), which is required for the stability and assembly of the gp91^*phox*^–p22^*phox*^ heterodimer (8, 9, 10, 11, 12, 13, 14, 15, 16). Rare hemizygous variants of *CYBB* are the most common genetic cause of CGD, underlying the X-linked recessive form of CGD (XL-CGD) (OMIM #306400), which accounts for approximately two thirds of cases worldwide (3, 8, 17, 18, 19, 20, 21). As a result, 70–80% of affected individuals are male. The remaining autosomal recessive (AR) forms are caused by rare homozygous or compound heterozygous variants of *CYBA* (OMIM #608508), *NCF1* (OMIM #608512), *NCF2* (OMIM #608515), *NCF4* (OMIM #613960), and *CYBC1* (OMIM #618334) (1, 8, 10, 11, 12, 13, 22, 23, 24, 25). The incidence of CGD has been estimated at 1 per 200,000–250,000 live births and varies between countries worldwide (19, 20, 26, 27). However, the true incidence of CGD in Colombia remains unclear due to the absence of large, systematically characterized cohorts. Several major contributions from Latin America, including efforts to establish registries in multiple countries, have expanded regional knowledge of the disease, but specific data for Colombia remain limited and are derived primarily from isolated case reports and small registry-based series (3, 18, 28, 29, 30). This study addressed this gap in our knowledge, through characterization of the clinical, microbiological, and genetic features of Colombian patients with CGD.

## Results

### Demographic and genetic analysis of CGD patients in Colombia

**Figure 1.**
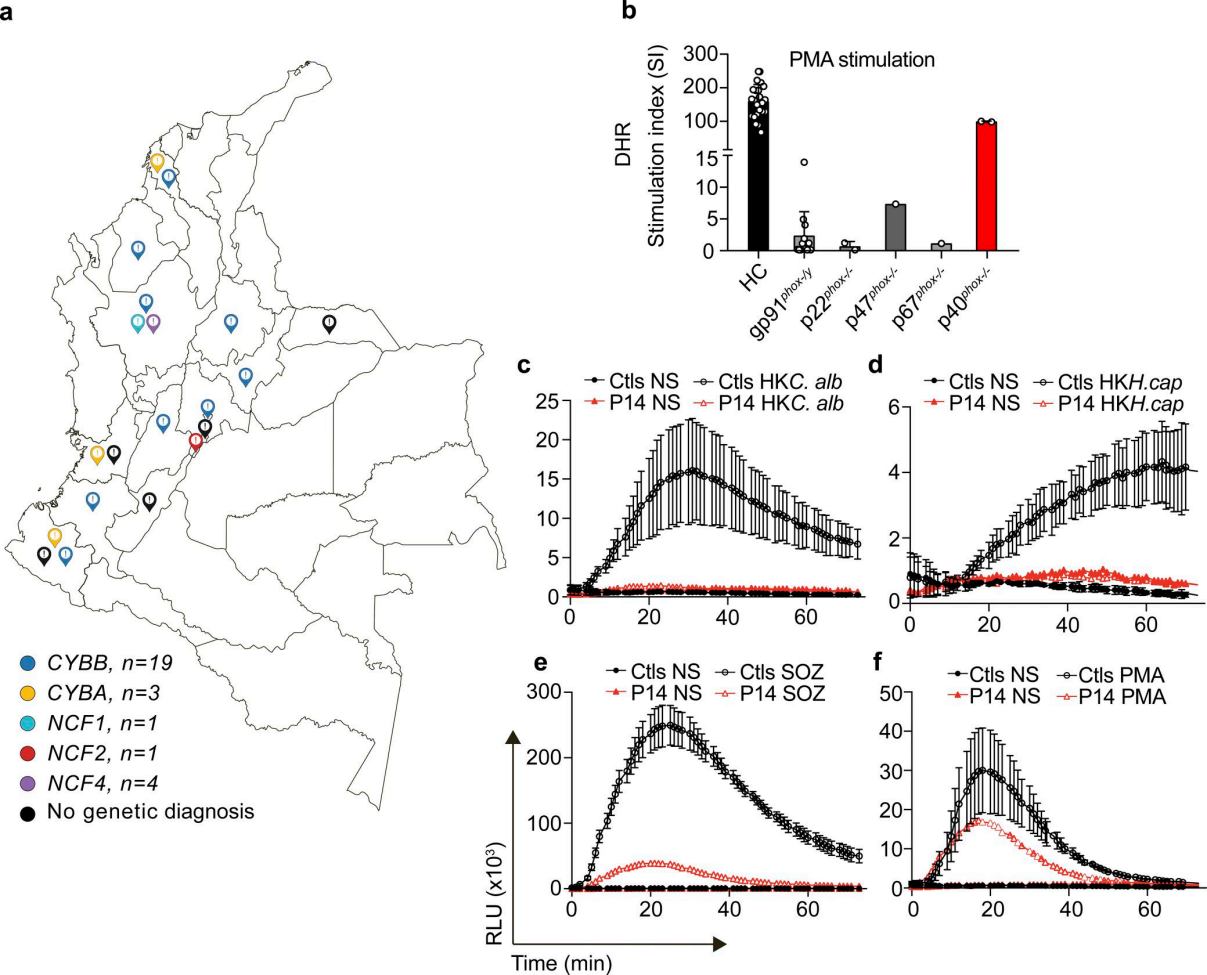
**Geographic distribution and impaired NADPH oxidase activity in Colombian patients with CGD. (a)** Geographic distribution of CGD cases in Colombia. **(b)** DHR oxidation levels in Colombian CGD patients (gp91^*phox*^, *n* = 13; p22^*phox*^, *n* = 2; p40^*phox*^, *n* = 2; p47^*phox*^, *n* = 1; and p67^*phox*^, *n* = 1), including the preserved PMA-induced activity of neutrophils seen in neutrophils of two patients with p40^*phox*^ deficiency. **(c–f)** In patient P14 with the p40^*phox*^ deficiency, neutrophil superoxide generation was measured over a time course of 60 min in response to serum-opsonized heat-killed *C. albicans* (HK*C.alb*) (c), heat-killed *H. capsulatum* (HK*H.cap*) (d), serum-opsonized zymosan (SOZ) (e), and PMA (f). The errors bars indicate standard deviation (SD) in panels b–e. HC, healthy control; NS, non stimulated.

We investigated 39 individuals from 32 unrelated Colombian kindreds enrolled in this study (Table 1 and Fig. 1 a). The patients originated from 12 different departments of Colombia: Cundinamarca (*n* = 13), Antioquia (*n* = 6), Nariño (*n* = 3), Valle del Cauca (*n* = 2), Bolívar (*n* = 2), Tolima (*n* = 2), and Cauca, Huila, Arauca, Córdoba, Boyacá, and Santander (*n* = 1 each). The regional origin of five patients was not specified (Fig. 1 a). 35 patients (89.74%) were male and four (10.25%) were female. Consanguinity was documented in two patients (P8 and P19), and one patient belonged to an endogamic community (P10); all three individuals had AR-CGD (Table 1). The median age at symptom onset was 3 months old (mo) (interquartile range [IQR] 1–8.5), and median age at CGD diagnosis was 23 mo (IQR 6.5–39). Whole-exome sequencing (WES) or Sanger sequencing was performed on the probands whenever feasible (Table 1). Eight patients did not undergo genetic testing and were diagnosed on the basis of clinical manifestations and a DHR assay performed several years earlier (Table 1). 22 patients carried hemizygous *CYBB* variants, including hemizygous missense (*n* = 3), nonsense (*n* = 11), essential splice site (*n* = 4), and large deletion variants (*n* = 4). Most of these variants have already been reported, but three of the *CYBB* were new: c.563T>G predicted p.L188*, c.935T>A predicted p.M312K, and c.1314 + 1_+5del. We also identified one female patient (P32) with a *CYBB* defect displaying skewed X inactivation (c.1226C>T; p.A409E). Three patients (7.7%) were homozygous (essential splice site) or compound heterozygous (small deletion and essential splice site) for pathogenic variants of *CYBA*, including the previously unreported c.59-1G>A variant. One patient was homozygous for the very common pathogenic variant c.75_76del (often written c.75_76delGT) of *NCF1*, consistent with AR-CGD. One patient was homozygous for a previously reported pathogenic variant of *NCF2* (c.233G>A; p.G78Q), associated with AR-CGD. Finally, one patient (P14) was homozygous for a variant of *NCF4* also carried by three asymptomatic siblings (P12, P13, and P15) in the homozygous state (Table 1) (9). These findings highlight the predominance of XL-CGD in this Colombian cohort, the broad geographic distribution of affected families, and the presence of both classic AR forms and a separate subgroup carrying *NCF4* variants.

**Table 1.** Overview of the clinical, genetic, and microbiologic features of Colombian CGD patients

Kindred	Pt	Sex	Gene	Variant/predicted protein	Age at onset (months)	Age at CGD diagnosis (months)	Site/type of infection	Microorganisms	Noninfectious manifestations	Outcome
A	P1	M	*CYBB*	c.809 G>A/y (p.W270*/y)	14	24	Lymphadenitis, pneumonia, bacteremia, abscess, and BCG-itis	*S. aureus*, *Salmonella* spp., *Klebsiella ozaenae*, and *M. bovis*–BCG	None	Alive after HSCT
B	P2	M	*CYBB*	c.563T>G/y (p.L188*/y)	1	12	Pneumonia, bacteremia, and pulmonary nodes	*Salmonella* spp., *E. coli*, *Pseudomonas* spp., and *B. cepacia*	Inflammatory bowel disease	Alive after HSCT
	P3	M	*CYBB*	c.563T>G/y (p.L188*/y)	1	10	Lymphadenitis, pneumonia, and bacteremia	*S. aureus* and *Klebsiella* spp.	Granulomas and inflammatory bowel disease	Alive after HSCT
C	P4	M	*CYBB*	c.919del/y (p.T307Pfs*6/y)	1	27	Lymphadenitis, pneumonia, bacteremia, osteomyelitis, and abscess	*S. aureus*, *Serratia* spp., and *Salmonella* spp.	None	Died due to *Salmonella* sepsis
	P5	M	*CYBB*	c.919del/y (p.T307Pfs*6/y)	1	48	Lymphadenitis, neonatal sepsis, pneumonia, bacteremia, and abscess	*S. aureus* and *Salmonella* spp.	None	Died due to *Salmonella* sepsis
	P6	M	NS	NS	7	84	Lymphadenitis, pneumonia, and bacteremia	*S. aureus*, *E. coli*, *Salmonella* spp	Autoimmune cytopenia	Died due to *Salmonella* sepsis
D	P7	M	*CYBB*	c.935T>A/y (p.M312K/y)	1	2	Recurrent pneumonia, urinary tract infections, and gastroenteritis	*Klebsiella* spp. and respiratory syncytial virus	None	Died after HSCT due to GI infection, graft failure
E	P8	F	*NCF1*	c.75_76del/c.75_76del (p.Y26Hfs*26/p.Y26Hfs*26)	96	132	Recurrent abscesses (liver, lung, and skin), pneumonia, lymphadenitis, and chronic diarrhea	*S. aureus* and *Aspergillus* spp.	Eczema	Alive with prophylaxis
F	P9	F	*CYBA*	c.59-1G>A/c.177del	1	15	Lymphadenitis, pneumonia, recurrent abscesses (submandibular, skin, legs, arms, and neck), osteomyelitis, chronic diarrhea, and septic arthritis	Non-typhi *Salmonella*	Granulomas	Alive after HSCT. Thyroid tumor after HSCT
G	P10	M	*CYBA*	c.177del/c.177del (p.K60Rfs*14/p.K60Rfs*14)	2	4	Lymphadenitis, pneumonia, bacteremia, sepsis, and BCG-osis	*M. bovis*–BCG	Granulomas	Died after HSCT due to sepsis and BCG-osis
H	P11	M	*CYBB*	c.736C>T/y (p.Q246*/y)	2	2	Pneumonia, urinary tract infections, bronchiolitis, and acute otitis media	*C. tropicalis* and *Enterococcus* spp.	None	Alive with prophylaxis
I	P12 (Ref 9)	M	*NCF4*	c.172C>T/c.172C>T (p.R58C/p.R58C)	0	120	Asymptomatic	None	None	Alive without prophylaxis
	P13 (Ref 9)	F	*NCF4*	c.172C>T/c.172C>T (p.R58C/p.R58C)	0	96	Asymptomatic	None	None	Alive without prophylaxis
	P14 (Ref 9)	M	*NCF4*	c.172C>T/c.172C>T (p.R58C/p.R58C)	36	36	Disseminated histoplasmosis (lymphadenitis, liver, spleen, serum, urine, and BM), tracheitis, perforated appendicitis, bacteremia, and chronic diarrhea	*H. capsulatum*, *Klebsiella pneumoniae*, and parainfluenza virus	None	Alive without prophylaxis
	P15 (Ref 9)	M	*NCF4*	c.172C>T/c.172C>T (p.R58C/p.R58C)	0	36	Asymptomatic	None	None	Alive without prophylaxis
J	P16	M	*CYBB*	g. (?_30653423)_(38220871_?)/y	1	8	Multilobar pneumonia, bronchiolitis, cellulitis, BCG-itis, and bacteremia	*B. cepacia*, *Aspergillus* spp., and *M. bovis*–BCG	Granulomas, Duchenne muscular dystrophy	Alive after second HSCT
K	P17	M	*CYBB*	c.1314 + 1_+5del/y	1	36	Pneumonia, recurrent gastroenteritis, hepatic, and cutaneous abscesses	*S. marcescens*	None	Died
L	P18	M	NS	NS	1	1	Neonatal sepsis, pneumonia, and bacteremia	*Aspergillus* spp. and *Klebsiella* spp.	None	Died after HSCT due to multiorgan dysfunction
M	P19	M	NS	NS	6	6	Pneumonia, bacteremia, and liver abscesses	*S. marcescens*	None	Died due to *S. marcescens* sepsis
N	P20	M	*CYBB*	c.15del/y (p.V6*/y)	4	6	Lymphadenitis, pneumonia, bacteremia, and abscess	*S. aureus*, *S. marcescens*, and *Salmonella* spp.	None	Died
	P21	M	*CYBB*	c.15del/y (p.V6*/y)	7	24	Hepatic abscess and osteomyelitis	*S. marcescens*	None	Alive with prophylaxis
O	P22	M	*CYBA*	c.58 + 4_58+7del/c.58 + 4_58+7del	1	7	Lymphadenitis, pneumonia, bacteremia, and BCG-itis	*P. fluorescens*, *P. putida*, *B. cepacia*, and *M. bovis*–BCG	Autoimmune cytopenia, granulomas, and cow’s milk allergy	Alive after HSCT
P	P23	M	*CYBB*	c.805-2A>T/y	3	12	Lymphadenitis, pneumonia, bacteremia, and BCG-itis	*E. cloacae*, *Salmonella* spp., and *M. bovis*–BCG	Colitis	Died after HSCT due to severe GVHD and infection
Q	P24	M	NS	NS	5	23	Lymphadenitis, pneumonia, bacteremia, and osteomyelitis	*A. baumannii*, *Pseudomonas* spp., and *Aspergillus* spp.	Infectious secondary hemophagocytic lymphohistiocytosis	Died after HSCT and lymphoproliferative disorder
R	P25	M	*CYBB*	c.565_568del/y (p.I189Sfs*24/y)	1	8	Neonatal sepsis, pneumonia, and bacteremia	*S. aureus* and *Aspergillus* spp.	Granulomas	Alive after HSCT
S	P26	M	*CYBB*	c.676C>T/y (p.R226*/y)	6	6	Pneumonia and bacteremia	*S. marcescens* and *Salmonella* spp.	Inflammatory bowel disease	Alive after second HSCT
T	P27	M	NS	NS	10	14	Lymphadenitis, pneumonia, bacteremia, and abscess,	*S. aureus* and *Salmonella* spp.	None	Died due to sepsis
U	P28	M	*NCF2*	c.233G>A/c.233G>A (p.G78Q/p.G78Q)	60	180	Lymphadenitis, pneumonia, bacteremia, and abscess	*S. aureus*, *Salmonella* spp., *M. pneumoniae*, and *M. tuberculosis*	Granulomas	Alive with prophylaxis
V	P29	M	NS	NS	7	48	Neonatal sepsis, lymphadenitis, pneumonia, bacteremia, and abscess	*S. aureus*, *S. marcescens*, *Pseudomonas* spp., and *L. pseudomesenteroides*	Autoimmune cytopenias	Died due to sepsis
W	P30	M	*CYBB*	c. (?_−54)_(653_?)/y	3	3	Pneumonia, bacteremia, abscess, BCG-itis, and urinary tract infection	*S. aureus*, *Aspergillus* spp., and *M. tuberculosis*	Inflammatory bowel disease, progressive muscle weakness, and Duchenne muscular dystrophy	Died after HSCT due to severe GVHD
	P31	M	*CYBB*	c. (?_−54)_(653_?)/y	12	42	Lymphadenitis, pneumonia, osteomyelitis, liver abscess, and BCG-itis	*S. aureus*, *M. bovis*–BCG, *Salmonella* spp., *E. coli*, and *Pseudomonas* spp.	Inflammatory bowel disease, progressive muscle weakness, and Duchenne muscular dystrophy	Died due to infectious complications
X	P32	F	*CYBB*	c.1226C>T/WT (p.A409V/WT)	36	144	Lymphadenitis, recurrent pneumonia, bacteremia, and abscess	*S. aureus*, *M. tuberculosis*, *B. cepacia*, and *Aspergillus* spp.	None	HSCT with graft failure and autologous reconstitution, alive with prophylaxis
Y	P33	M	NS	NS	48	60	Lymphadenitis, pneumonia, bacteremia, osteomyelitis, and liver abscess	*Aspergillus* spp., *Pseudomonas* spp., and *M. tuberculosis*	Granulomas	Died due to invasive aspergillosis after HSCT
Z	P34 (31)	M	*CYBB*	c.46-14−11delTTCT insGAA/y	2	48	Recurrent diarrhea, recurrent cutaneous and perianal abscess, and abscess	*Salmonella* spp.	Inflammatory bowel disease and colitis	Unknown
AA	P35 (32)	M	*CYBB*	g.(? _37780078)_(37810917A_?)/y	7	132	Lymphadenitis, bacteremia, perianal abscess, meningitis, and BCG-itis	*B. cepacia*, *Salmonella* spp., and *M. bovis*–BCG	None	Alive after HSCT
AB	P36 (Ref 33)	M	*CYBB*	c.1587-2A>G/y	12	Unknown	Lymphadenitis, osteomyelitis, bacteremia, and sepsis	*Klebsiella* spp. and *Salmonella* spp.	None	Died
AC	P37 (Ref 33)	M	*CYBB*	c.271C>T/y (p.R91*/y)	Unknown	Unknown	Lymphadenitis, osteomyelitis, adenitis, meningitis, bacteremia, and sepsis	*Salmonella* spp.	None	Died
AD	P38 (Ref 33)	M	*CYBB*	c.674A>T/y (p.E225V/y)	3	Unknown	Cervical suppurative lymphadenitis and sepsis	*S. aureus*	None	Died
AE	P39	M	NS	NS	Unknown	36	Mediastinal lymphadenitis, recurrent pneumonia, bacteremia, and thoracic wall abscess	*Salmonella* spp. and *Aspergillus* spp.	None	Alive with prophylaxis, awaiting HSCT

BCG, Bacillus Calmette–Guérin; BM, bone marrow; F, female; GI, gastrointestinal; M, male; NS, not studied; Pt, patient; Ref, references in which the patients were previously reported.

### Impaired NADPH oxidase activity of neutrophils

The DHR assay, which measures phorbol 12-myristate 13-acetate (PMA)–induced ROS production by neutrophils, is the standard functional test for the diagnosis of classic CGD (9, 34). The percentage DHR oxidation was determined for all patients (Fig. 1 b, data not shown in 20 patients) and was zero or markedly lower than that in healthy controls in 35 CGD patients; this assay also revealed the distinctive profile of p40^*phox*^ deficiency, with DHR activity in response to PMA stimulation remaining normal in four related individuals from kindred I (P12, P13, P14, and P15) (9). Impaired activation was observed when neutrophils from P14 and P12 were primed with low concentrations of tumor necrosis factor (TNF) and then stimulated with N-formyl-Met-Leu-Phe (fMLP) (data not shown). A more detailed assessment of intracellular superoxide production in P14 revealed a marked decrease following stimulation with serum-opsonized heat-killed *Candida albicans* (Fig. 1 c), heat-killed *Histoplasma capsulatum* (Fig. 1 d), or serum-opsonized zymosan (Fig. 1 e), whereas PMA-induced superoxide production remained relatively normal (Fig. 1 f). Together, these findings confirm that all Colombian patients with variants of *CYBB*, *CYBA*, *NCF1*, and *NCF2* display impaired neutrophil NADPH oxidase–dependent responses, consistent with a diagnosis of CGD, except for those with the *NCF4* variant, who displayed impaired ROS production in response to physiological stimuli.

### Clinical disease of Colombian CGD patients

**Figure 2.**
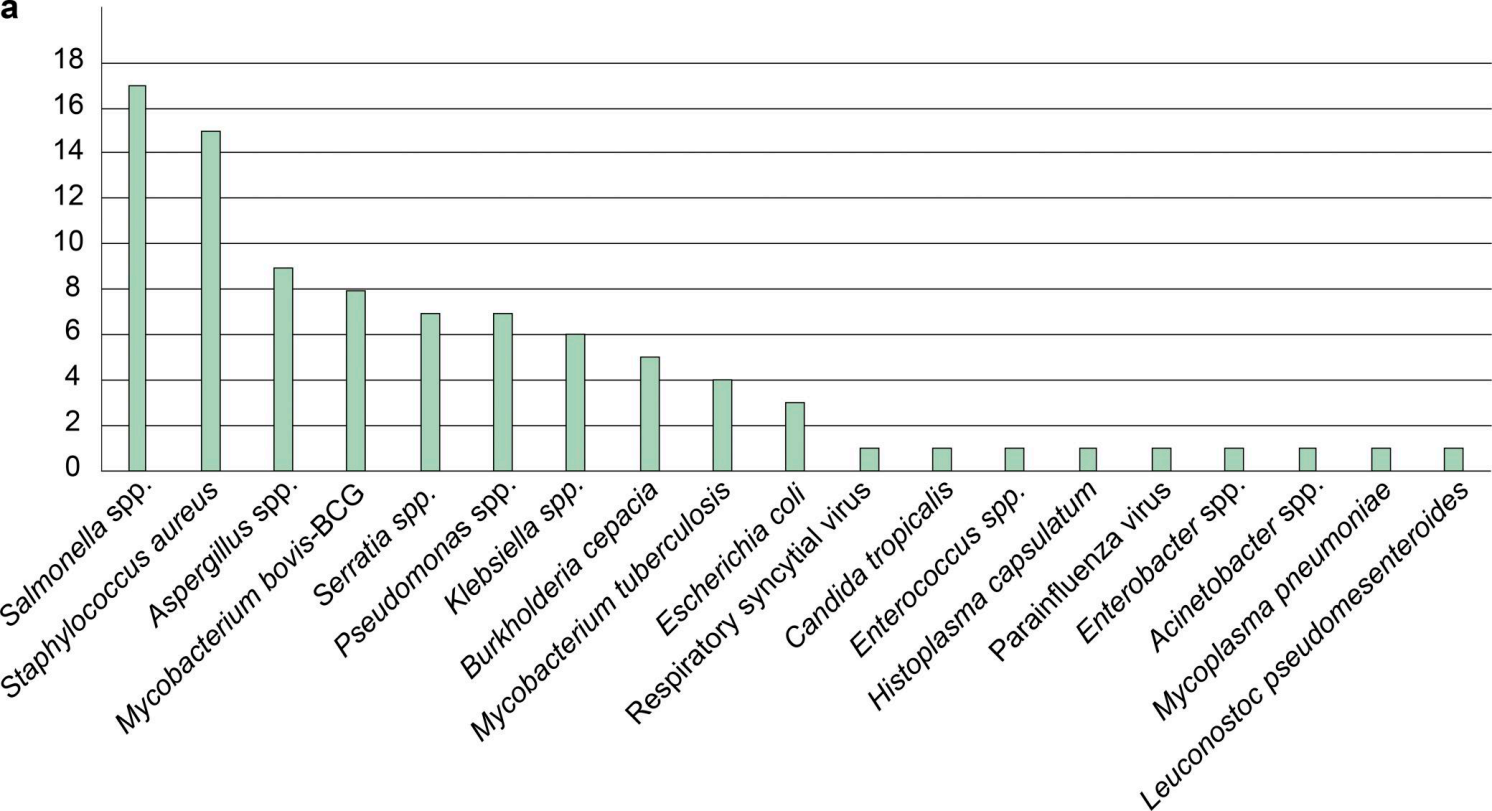
**Infectious disease in Colombian patients with CGD. (a)** Number of microorganisms isolated from documented infections. The y-axis represents the number of patients with confirmed infection caused by each microorganism.

Bacterial infections were the most common manifestations. *Salmonella* spp. isolation was reported for 17 patients (43.58%), with bacteremia documented in 12 of these patients (70.58%). Single cases of pulmonary nodules, brain abscess, lymphadenitis, and septic arthritis and three cases of sepsis were also observed (Table 2). *Klebsiella* spp., a member of the Enterobacteriaceae family like *Salmonella*, was identified in six infection cases (bacteremia, osteomyelitis, and pneumonia) (35). *S. aureus* was isolated from 15 patients (38.46%; Table 1). Other Gram-negative bacteria were also found (Fig. 2), including *Pseudomonas* spp., *Pseudomonas fluorescens*, *Pseudomonas putida*, *B. cepacia*, *S. marcescens*, *Acinetobacter baumannii*, and *Enterobacter cloacae*. We also observed several patients infected with another Gram-positive coccus, such as *Enterococcus* spp., or *Leuconostoc pseudomesenteroides*. Bacterial infections of the lungs (pneumonia, *n* = 29; 74.35%) were the most frequent, followed by bacteremia (*n* = 27; 69.23%), abscesses (*n* = 19; 48.72%), lymphadenitis (*n* = 18; 46.15%), and osteomyelitis (*n* = 8; 20.51%; Table 1). Clinical manifestations were compatible with CGD in 36 of the 39 patients described in this manuscript, with the other three individuals with *NFC4* variants remaining asymptomatic to date (P12, P13, and P15). All patients were vaccinated with a single dose of *M. bovis*–BCG (strain Pasteur 1173), administered to the left arm, during the neonatal period or within the first month of life. Eight patients (P1, P10, P16, P22, P23, P30, P31, and P35; 17.95%) developed axillary lymphadenitis following vaccination (BCG-itis), progressing to disseminated BCG disease (BCG-osis) in one patient (P10). Pulmonary infection with *M. tuberculosis* was identified in four patients (P28, P30, P32, and P33) among these patients, only P30 also developed BCG-itis.

**Table 2.** *Salmonella* infections in CGD patients

Patient	Isolate name	Method	Site/type of infection
P1	*Salmonella* spp.	Culture	Bacteremia
P2	*Salmonella* spp.	Culture	Bacteremia and pulmonary node
P4	*Salmonella* spp.	Culture	Sepsis
P5	*Salmonella* spp.	Culture	Sepsis and brain abscess
P6	*Salmonella* spp.	Culture	Sepsis
P9	Non-typhi *Salmonella*	Culture	Bacteremia and arthritis
P20	*Salmonella* spp.	Culture	Bacteremia
P23	*Salmonella* spp.	Culture and PCR	Bacteremia and colitis
P26	*Salmonella* spp.	Culture	Bacteremia
P27	*Salmonella* spp.	Culture	Lymphadenitis
P28	*Salmonella* spp.	Culture	Bacteremia
P31	*Salmonella* spp.	Culture	Bacteremia
P34	*Salmonella* spp.	Culture	Colitis
P35	*Salmonella* spp.	Culture	Bacteremia
P36	*Salmonella* spp.	Culture	Bacteremia
P37	*Salmonella* spp.	Culture	Bacteremia
P39	*Salmonella* spp.	Culture	Bacteremia

Nine patients (P8, P16, P18, P24, P25, P30, P32, P33, and P39; 23.08%) developed aspergillosis, and two others presented other fungal infections (*Candida tropicalis*, *n* = 1; *H. capsulatum*, *n* = 1). Two patients had documented viral infections (respiratory syncytial virus and parainfluenza) with moderate clinical manifestations, and one patient was infected with *M. pneumoniae* (Fig. 2 and Table 1). Granulomas were observed in eight patients (20.51%), inflammatory bowel disease in six patients (15.38%), autoimmune cytopenia in three patients (7.69%), and a macrophage activation syndrome secondary to infection was documented in one patient (2.56%; Table 1). Patients P16, P30, and P31 also presented progressive muscle weakness, consistent with the large deletion on the X chromosome also encompassing the *DMD* gene responsible for Duchenne muscular dystrophy. Together, these findings highlight the substantial infectious and inflammatory burden of CGD in these Colombian patients, while underscoring the clinical heterogeneity associated with different genetic defects.

### Treatment of CGD patients

All but four (p40^*phox*^ deficiency) of the patients received prophylaxis with trimethoprim-sulfamethoxazole and antifungal drugs, including itraconazole (*n* = 21, 53.84%), voriconazole (*n* = 13, 33.3%), or posaconazole (*n* = 3, 7.69%). 16 patients (41.03%) received human recombinant interferon-γ (hrIFN-γ) treatment in addition to antimicrobial therapy. 17 patients underwent hematopoietic stem cell transplantation (HSCT, 43.6%). Three patients (P16, P26, and P33) required a second HSCT, for a total of 20 procedures (*n* = 20). Median age at HSCT was 28 mo (IQR 11–74).

Two patients are currently awaiting HSCT. The donor was haploidentical, with posttransplantation cyclophosphamide in 11 transplants (55%) and umbilical cord blood (UCB) in eight transplants (40%), and one patient underwent HSCT from a matched sibling donor (5%) (Table 3). Myeloablative conditioning was used in seven transplants and reduced-intensity conditioning in 13 (65%). 16 of the transplant recipients received a busulfan and fludarabine conditioning regimen, associated with cyclophosphamide in three cases and melphalan in two (Table 3). Three transplant recipients received a busulfan-cyclophosphamide conditioning regimen, and four underwent total body irradiation. All patients received antithymocyte globulin for in vivo T cell depletion to reduce the incidence of graft-versus-host disease (GVHD) and graft failure. Graft failure occurred in six transplants (30%): five receiving cells from a haploidentical donor and one from UCB. Acute (a)-GVHD developed in eight transplant recipients: a-GVHD I (*n* = 1), a-GVHD II (*n* = 2), a-GVHD III (*n* = 2), and a-GVHD IV (*n* = 3). Chronic (c)-GVHD occurred in seven transplant recipients: c-GVHD I (*n* = 2), c-GVHD II (*n* = 3), and c-GVHD III (*n* = 2). One patient (P9) developed a thyroid tumor, and one patient (P24) presented posttransplant lymphoproliferative disease. Overall, these data reflect the heterogeneous therapeutic approaches used in this cohort, including broad antimicrobial prophylaxis and hrIFN-γ, and underscore the central but challenging role of HSCT, with the use of various conditioning strategies, frequent GVHD, and marked graft failure rates, in the management of CGD patients.

**Table 3.** Overview of HSCT in the cohort of Colombian CGD patients

Pt	Age at HSCT (months)	Transplant source and match	Conditioning	Regimen	Graft failure	a-GVHD	c-GVHD	Survival	Complications and follow-up after HSCT
P1	173	UCB 5/6	RIC	Busulfan/cyclophosphamide	No	II	II	Yes	Alive 11 years after HSCT
P2	32	UCB 5/6	RIC	Busulfan/fludarabine	No	No	No	Yes	Alive 8 years after HSCT
P3	56	UCB 5/6	RIC	Busulfan/cyclophosphamide	No	III	No	Yes	Alive 6 years after HSCT
P7	11	UCB 5/6	MAC	Busulfan/fludarabine	No	No	No	No	Died after HSCT due to GI infection, graft failure
P9	16	Haplo-PtCy	MAC	Busulfan/fludarabine/cyclophosphamide/TBI	Yes	I	I	Yes	Alive 9 years after HSCT. Thyroid tumor after HSCT
P10	5	Haplo-PtCy	MAC	Busulfan/fludarabine/cyclophosphamide/TBI	No	No	III	No	Alveolar hemorrhage. Died due to severe GVHD and sepsis, BCG-osis
P16	6	Haplo-PtCy	MAC	Busulfan/fludarabine	Yes	NE	NE	NE	Graft failure, underwent second HSCT
	7	Haplo-PtCy	MAC	Busulfan/fludarabine/cyclophosphamide/TBI	No	No	No	Yes	Alive 9 years after HSCT
P18	71	Haplo-PtCy	RIC	Busulfan/fludarabine	NE	NE	NE	No	Early death after HSCT, multiorgan dysfunction
P22	11	Haplo-PtCy	RIC	Busulfan/fludarabine	No	No	No	Yes	Alive 11 mo after HSCT
P23	20	UCB 5/6	RIC	Busulfan/fludarabine	No	IV	II	No	Died due to severe GVHD and infection
P24	34	UCB 5/6	RIC	Busulfan/fludarabine	No	IV	III	No	Died due to lymphoproliferative disorder 3 years after HSCT, Burkitt lymphoma EBER-positive
P25	7	Haplo-PtCy	MAC	Busulfan/fludarabine/melphalan	No	II	I	Yes	Laryngomalacia and tracheal granuloma. Alive 21 mo after HSCT
P26	24	Haplo-PtCy	RIC	Busulfan/fludarabine	Yes	NE	NE	NE	Graft failure, underwent second HSCT
	36	Haplo-PtCy	RIC	Busulfan/cyclophosphamide	No	III	II	Yes	*Pseudomonas mendocina* bacteremia. Probable lung aspergillosis. *Clostridium difficile* gastrointestinal infection. Gastrointestinal bleeding. Alive 1.7 years after HSCT
P30	14	UCB 5/6	RIC	Busulfan/fludarabine	No	IV	NE	No	Died due to severe GVHD
P32	132	UCB 5/6	RIC	Busulfan/fludarabine	Yes	NE	NE	Yes	Graft failure. Alive with prophylaxis
P33	75	Haplo-PtCy	RIC	Busulfan/fludarabine/melphalan	Yes	NE	NE	NE	Graft failure, underwent second HSCT
	90	Haplo-PtCy	MAC	Busulfan/fludarabine	Yes	NE	NE	No	Died after HSCT due to invasive aspergillosis and multiorgan failure
P35	96	Matched sibling donor	RIC	Fludarabine/cyclophosphamide/TBI	No	No	No	Yes	Alive after HSCT

a-GVHD, acute graft vs. host disease; c-GVHD, chronic graft vs. host disease; EBER, Epstein-Barr virus-encoded small RNAs; GI, gastrointestinal; Haplo-PtCy, haploidentical hematopoietic stem cell transplantation with posttransplant cyclophosphamide; MAC, myeloablative conditioning; NE, not evaluated; Pt, patient; RIC, reduced intensity conditioning; TBI, total-body irradiation.

### Mortality and survival

**Figure 3.**
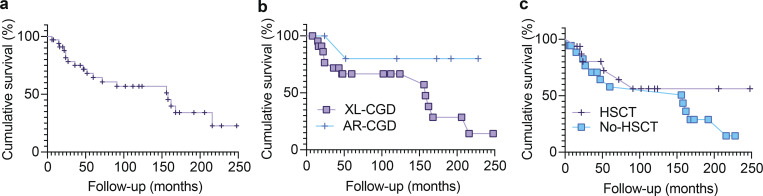
**Survival in patients with CGD. (a)** Kaplan–Meier survival curve showing cumulative overall survival of CGD patients during follow-up (up to ∼250 mo). The y-axis represents cumulative survival (%), and the x-axis indicates follow-up time in months. **(b)** Survival according to inheritance pattern (XL-CGD vs. AR-CGD). Kaplan–Meier survival analysis comparing cumulative survival between patients with XL-CGD and AR-CGD. **(c)** Survival according to HSCT status. Kaplan–Meier survival curves comparing cumulative survival between CGD patients who underwent HSCT and those who did not. The y-axis represents cumulative survival (%), and the x-axis indicates follow-up time in months (up to ∼250 mo).

In total, 19 patients diagnosed with CGD died, including 12 patients with XL-CGD and one patient with AR-CGD; in six patients, the genetic defect was not identified. Fig. 3 presents overall survival (Fig. 3 a), differential survival for patients with XL-CGD versus AR-CGD (Fig. 3 b), and survival for patients who underwent HSCT compared to those who did not (Fig. 3 c). The leading cause of death was infectious complications, which occurred in 12 patients who had not undergone HSCT and seven patients who had undergone HSCT. The causes of death in the HSCT group included posttransplant infections (*n* = 2), combined GVHD and infection (*n* = 2), severe a-GVHD (*n* = 1), multiorgan failure (*n* = 1), and posttransplant lymphoproliferative disease (*n* = 1). All individuals with AR p40^*phox*^ deficiency (one symptomatic and three asymptomatic) remain alive (Table 1). In the 36 affected patients, excluding the three asymptomatic individuals with *NCF4* variant, overall survival was higher in patients who underwent HSCT (*n* = 10 of 17 HSCT, 58.8%) than in those who did not (*n* = 6 of 18 non-HSCT, 33.3%). Median survival was similar for AR-CGD and XL-CGD patients (log-rank chi-square 2.822, P = 0.093). Hazard ratio was higher for XL-CGD 4.857 (95% confidence interval [CI] 1.46–16.11). Mortality was higher in patients with XL-CGD (12 out of 23, 52%) than in AR-CGD (1 out of 6, 17%), but it did not reach statistical significance (Fisher’s exact test P = 0.1834).

Median survival was similar for patients who underwent HSCT compared to those that did not (log rank chi-square 0.577, P = 0.448). Hazard ratio was 0.685 (95% CI 0.269–1.747). Mortality was higher in patients who did not undergo HSCT (12 out of 18, 67%) than in those who did (6 out of 16, 37.5%), but it did not reach statistical significance (chi-square 2.892, P = 0.0890). These findings highlight the high mortality burden associated with CGD in this cohort, together with the potential survival benefit of HSCT despite its inherent risks.

## Discussion

We report the first comprehensive cohort of Colombian patients with CGD. The geographic distribution of patients across 12 departments likely reflects referral patterns rather than true national prevalence. Geographically remote areas, particularly along the Andes mountain range and in southeastern jungle regions such as the Amazon and Orinoquía, are probably underrepresented due to barriers in healthcare access, limited specialist availability, and long travel distances. Genetic analyses demonstrated that XL-CGD was the most prevalent form, consistent with previous reports for Latin-American, Mexican, and U.S. cohorts, but contrasting with reports from Egypt, China, Morocco, and Turkey, where AR-CGD predominates (18, 23, 28, 29, 30, 36, 37). Notably, we identified variants not yet described in *CYBB*, specifically c.563T>G (predicted p.L188*), c.935T>A (predicted p.M312K), and c.1314 + 1_+5del, expanding the spectrum of known CGD-causing variants. We also identified a female patient (P32) heterozygous for the p.A409E variant with clinical CGD, highly impaired ROS production, and skewed X-chromosome inactivation on whole blood. Three patients with gp91^*phox*^ deficiency displayed a combined CGD–Duchenne muscular dystrophy (DMD) syndrome; in one case, the diagnosis of DMD was established only after HSCT. This dual phenotype is biologically and clinically plausible because *CYBB* and *DMD* are located in close proximity on Xp21.1, where large deletions can disrupt both genes simultaneously (38). Nine of the 39 patients (23%) had AR-CGD. Among them, three individuals carrying *NCF4* variants are asymptomatic to date; therefore, these three individuals were excluded from the clinical and survival analyses. Other Latin American studies have found AR-CGD in 13–33% of cases, but in countries with a higher incidence of consanguineous marriages, this form can account for up to 60% of CGD cases (18, 23, 27, 30, 36, 39, 40, 41, 42, 43). The frequency of p47^*phox*^ deficiency in our cohort was 3.2% (1/39), which is lower than the 24.7% (71/287) reported by Kuhns et al. in North American and European populations (44) but closer to the 7.6% (18/238) described by de Oliveira et al. in a more comparable regional context (18). Although p47^*phox*^ deficiency is often associated with a milder clinical course that could potentially delay diagnosis, our cohort represents one patient referred to a tertiary immunology center. Furthermore, given the modest sample size, proportional comparisons should be interpreted with caution. Our genetic studies identified one previously unreported *CYBA* variant, c.59-1G>A. Two patients carried the c.177del *CYBA* variant; one was homozygous (P10) and the other was compound heterozygous (P9) with the c.59-1G>A/c.177del variant. These two patients came from neighboring departments in Colombia, suggesting a possible local founder effect. Furthermore, we detected no pathogenic variants of *CYBC1*, consistent with its relative rarity in CGD cohorts worldwide (6, 45, 46, 47). P14 presented with severe infections and was diagnosed with *NCF4*-associated CGD. His three siblings, aged 3, 8, and 10 years at the time of his diagnosis, were all found to be homozygous for the same *NCF4* variant but have remained asymptomatic, demonstrating the variability of clinical expression of the disease, at least during adolescence. Continued longitudinal follow-up of the asymptomatic siblings will be essential to evaluate potential late-onset symptoms.

Median age at immunological diagnosis in our cohort was 23 mo, with an early median age at symptom onset, the main clinical manifestation being pneumonia, as reported in other studies (17, 18, 23, 27, 30, 36, 39, 40, 41, 42, 43). This probably reflects the predominance of XL-CGD, which is typically detected earlier in life and with more severe manifestations, together with ongoing improvements in the recognition of IEIs in Colombia. These improvements are being driven by increasing collaboration between clinical immunologists, infectious disease specialists, and pediatricians and by improvements in access to DHR testing and molecular diagnosis across the country. In our series, diseases due to *Salmonella* spp. were the most frequent infectious manifestation, consistent with findings from other Latin American, Mexican, and U.S. cohorts, but contrasting with the lower frequency of this infection reported in China, India, Turkey, Israel, Morocco, and Egypt (17, 18, 19, 21, 23, 27, 30, 36, 39, 40, 41, 42, 43). *Salmonella* spp. infection accounted for three deaths, highlighting the importance of this bacterium as a major clinical pathogen in patients with CGD in Colombia. Unfortunately, specific serotyping into typhoidal or non-typhoidal strains is not routinely performed in our clinical setting, limiting our ability to classify this severe infection. *Klebsiella* spp. infections were also frequent, consistent with the close phylogenetic and pathogenic similarities between *Klebsiella* and *Salmonella* (48, 49). Together, these findings highlight the need for early diagnosis, antimicrobial prophylaxis, and greater clinical suspicion of CGD, particularly in patients presenting with severe or recurrent *Salmonella* infections. One patient (P29) developed infection with *L. pseudomesenteroides*, an opportunistic microorganism mainly reported in hospitalized patients exposed to broad-spectrum antibiotics or invasive devices (50). No specific association with neutrophil defects has been established; therefore, this episode is more appropriately interpreted as potentially healthcare associated. All patients were vaccinated with BCG at birth, in line with Colombia’s universal immunization policy. Several patients were vaccinated despite a known family history of CGD, highlighting the need for the genetic counseling and explicit education of families concerning BCG-related risks. Confirmed or suspected *M. bovis*–BCG disease was observed in 17.95% of patients, a rate similar to that reported in Mexico, where this disease was observed in 30% of patients, but lower than that in the broader Latin American cohort (45%) (18, 30). *Aspergillus* spp. was identified as another important pathogen in our cohort, consistent with findings for other cohorts (18, 39, 45, 46, 47). Immune dysregulation was also observed, most notably inflammatory bowel disease in XL-CGD, consistent with the known inflammatory complications associated with this genotype (51). Overall mortality in our cohort exceeded 50%, but HSCT was associated with a marked improvement in survival (58.8 vs. 33.3%), confirming the curative benefits of this treatment, when available. The frequency of HSCT was higher in our series than in the most recently reported Latin American series (41 vs. 22%), although graft failure remained frequent in both series. In our cohort, the predominance of haploidentical and UCB transplants due to limited donor availability likely explains the increased graft failure incidence, rather than a population-specific biological effect. Reported graft failure rates after HSCT for CGD range from ∼5–20%, related to haploidentical or UCB grafts, particularly when reduced-intensity conditioning is used, low CD34^+^ cell dose, or significant pretransplant inflammation (52, 53, 54). In contrast, severe combined immunodeficiency generally demonstrates lower graft failure rates in matched donor settings, likely due to profound host immune deficiency facilitating engraftment (55, 56, 57). In our cohort, we observed marked clinical trends suggesting a worse prognosis for patients with the XL-CGD variant and for those who did not receive a HSCT. Although mortality was notably higher in patients without HSCT (67 vs. 37.5% in transplanted patients), this difference did not reach statistical significance (P = 0.089), likely due to the small sample size. However, it is important to highlight that the hazard ratio analysis did demonstrate that patients with XL-CGD have a significantly higher risk of mortality, almost five times higher, compared to the AR-CGD variant (Hazard Ratio 4.857; 95% CI 1.46–16.11). Altogether, these findings underscore the severity of the XL genotype and support the clinical benefit of HSCT, although multicenter studies with larger cohorts are required to statistically confirm the impact of transplantation on survival.

Expanding early access to immunological and genetic diagnosis, particularly in remote regions of Colombia, is essential to ensure timely referral for HSCT and to reduce preventable morbidity and mortality (18). Importantly, earlier diagnosis also increases the opportunities for genetic counseling, empowering families by helping them to understand inheritance patterns, reproductive risks, and preventive strategies, such as the avoidance of BCG vaccination in newborns at high risk. This study provides the first comprehensive characterization of CGD in Colombia, delivering crucial epidemiological and molecular insights that will enhance national diagnostic capacity and support better care for patients and their families across Colombia.

## Materials and methods

### Subjects

The patients and their relatives were living in Colombia (South America) and were followed up locally. They were recruited by the Group of IEIs (formerly the Primary Immunodeficiencies Group) in Medellin and at collaborating centers in Colombia. Written informed consent was obtained from the patients, their relatives, and the volunteer healthy controls enrolled in the study. Healthy volunteers were recruited at the University of Antioquia. This study was conducted in accordance with the “Scientific Standards for Technical and Administrative Health Research” established by Colombian Ministry of Health resolution 008430 of 1993. It was approved by the local review board of the University of Antioquia (F8790-07-0010) and by HOMI Fundación Hospital Pediátrico La Misericordia (735-24R). Experiments on samples from human subjects were performed in France and Colombia, in accordance with local regulations and with the approval of the corresponding institutional review boards.

### WES, sanger sequencing, and bioinformatic analysis

Genomic DNA (gDNA) was extracted with the Puregene DNA Purification Kit (Gentra Systems) from whole-blood samples collected from patients and probands. Exome capture was performed with Agilent SureSelect V4/V5/V6 kits (Agilent Technologies), and paired-end sequencing was performed on a HiSeq 4000 platform (Illumina) generating 100-base reads. The reads were aligned with the reference human genome GRCh38/hg38 with the Burrows-Wheeler Alignment tool (BWA v. 0.7.12-r1039) (58). PCR duplicates were removed with Picard tools (https://picard.sourceforge.net/). The GATK base quality score recalibrator was applied to correct sequencing artifacts. GATK HaplotypeCaller was used to identify variant calls (59, 60). In some cases, depending on the patient’s health insurance cover, genetic sequencing was performed by external laboratories. Variants were confirmed by Sanger sequencing on gDNA from patients, parents, and relatives, when possible. Amplicons were sequenced with BigDye Terminator technology and a 3500XL Genetic Analyzer Sequencer (Applied Biosystems). We used the software analyzer Genius R9 v9.1.3 (Snapgene, GSL Biotech LLC) and 4Peaks (Nucleobytes B.V.) to align the sequences obtained with the reference sequence deposited in the NCBI database.

### DHR assay

Whole blood from patients, unaffected relatives, and healthy controls was collected into heparin-containing tubes. Red blood cells were lysed with ammonium chloride solution. After washing, DHR (Sigma-Aldrich) and catalase (1,300 IU/ml; Sigma-Aldrich) were added, and the mixture was incubated at 37°C for 5 min. We then added buffered solution without stimulant or with PMA (400 ng/ml) (Sigma-Aldrich) to the tubes, which were incubated in a water bath at 37°C for 14 and 20 min, respectively. Samples were analyzed with a FACS SORT flow cytometer. We analyzed a total of 20,000 cells in the neutrophil gate (based on forward and side scatter) for fluorescence intensity in the FL-2 channel (61).

### Luminol assay

Luminol-enhanced chemiluminescence assays were performed on polymorphonuclear neutrophils (PMNs) to assess their NADPH oxidase activity. PMNs (2 × 10^5^ cells) were resuspended in PBS supplemented with glucose (PBS plus 0.9 mM CaCl_2_, 0.5 mM MgCl_2_, and 20 mM dextrose) and luminol (50 µM) and plated in 96-well plates in the presence or absence of superoxide dismutase (SOD; 75 µg/ml). Cells were incubated at 37°C for 10 min before the addition of PMA (400 ng/ml), opsonized heat-killed *C. albicans*, or 1 × 10^6^ heat-killed *H. capsulatum* cells. The number of relative light units (RLUs) was determined every 60–90 s over a period of 30 or 50 min with the long kinetic module in a Lmax microplate luminometer (Molecular Devices). Integrated RLU values were calculated with SoftMax software (Molecular Devices), with the subtraction of background chemiluminescence (measured in wells including all reagents but no cells) to obtain values for total ROS production.

### Statistical analysis

Statistical analyses were performed with IBM SPSS Statistics for Windows, Version 25.0 (IBM Corp.), and survival data were analyzed using the Kaplan–Meier method using GraphPad Prism version 11. Given the sample size, qualitative data are presented as frequencies and percentages, whereas quantitative data are reported as medians and IQR. Categorical variables were analyzed with chi-squared tests. P values below 0.05 were considered statistically significant.

## Ethics approval

Informed consent for participation in this study was obtained in accordance with local regulations, with approval from the Institutional Review Board (IRB). The experiments described here were performed in Colombia and in France, in accordance with local regulations and with the approval of the IRB of Necker Hospital for Sick Children, France, and the local review board of the Universidad de Antioquia (F8790-07-0010).

## Consent to participate

Written informed consent to participate was obtained from all the patients.

## Consent for publication

Consent to publish this report was obtained from patients. All the authors approved the final version of the manuscript.

## Data Availability

The data underlying this study are not publicly available due to patient privacy issues. The data are available from the corresponding author upon request.
